# Two-handed facemask technique effectively causes hyperventilation in electroconvulsive therapy: an observational study

**DOI:** 10.1186/s12871-022-01928-7

**Published:** 2022-12-05

**Authors:** Yoko Shimamoto, Michiyoshi Sanuki, Shigeaki Kurita, Masaya Ueki, Yoshie Kuwahara

**Affiliations:** Department of Anesthesiology, NHO Kure Medical Center, 3-1 Aoyama, Kure, Hiroshima, Japan

**Keywords:** Electroconvulsive therapy, Anesthesia, Hyperventilation, Facemask, Hypocapnia, Blood pressure

## Abstract

**Background:**

Electroconvulsive therapy (ECT) remains the mainstay treatment option for patients with psychiatric diseases, such as severe depression. Although various anesthetic techniques provide adequate therapeutic seizures, hyperventilation is a useful adjunct to augment seizure duration and improve seizure quality. We investigated how to efficiently use a facemask to accomplish protocolized hyperventilation and evaluate its effect on ECT seizure.

**Methods:**

We studied 60 patients aged ≥18 years who underwent ECT. The patients were divided into two groups according to the technique of facemask ventilation used: the one-handed (*n* = 30) and two-handed (n = 30) groups. Following anesthesia induction under preoxygenation conditions, hyperventilation induced hypocapnia in the one-handed facemask group with manual bag ventilation was compared to that in the two-handed facemask group with assisted pressure-controlled ventilation. Ictal and peri-ictal electroencephalogram parameters and cardiovascular responses were monitored and compared between the one-handed and two-handed groups.

**Results:**

The two-handed technique demonstrated better electroencephalogram regularity and minimized cardiovascular stress compared to the one-handed technique. These conclusions come from the fact that the one-handed technique induced a substantial volume of leaks around the facemask (201.7 ± 98.6 mL/breath), whereas minimal leaks (25.8 ± 44.6 mL/breath) with stabler and higher ventilation rate led to greater inhaled minute ventilation in the two-handed group (the one-handed group, 9.52 ± 3.94 L/min; the two-handed group, 11.95 ± 2.29 L/min; *p* <  0.005). At the end of ECT treatment, all parameters of blood pressure and heart rate increased significantly in both groups equally, with lower SpO_2_ and more ST-segment depression on the electrocardiogram in the one-handed group. Comparing baseline values before anesthesia, ECT treatment significantly depressed ST-segment in both groups, while the degree of depression in ST-segment increased significantly in the one-handed group compared to that in the two-handed group.

**Conclusions:**

End-tidal carbon dioxide monitoring for hyperventilation can reliably ensure hypocapnia only in the two-handed group. In ECT, the two-handed technique assisted by pressure-controlled ventilation is an effective and practical method for hyperventilation to induce adequate therapeutic seizures. While, the two-handed group with sufficient preoxygenation did not cause more cardiovascular stress than the one-handed group.

**Trial registration:**

UMIN Clinical Trials Registry 000046544, Date of registration 05/01/2022.

## Introduction

Electroconvulsive therapy (ECT) is a clinically proven procedure, wherein small electric currents pass through the brain and intentionally trigger a short seizure. These neurological alterations include neuronal stimulation and depolarization as well as changes in cerebral blood flow, regional metabolism, and the blood-brain barrier [1]. ECT is widely practiced, mainly to treat severe depression, and is also used in some patients with bipolar disorder, schizophrenia, catatonia, and neuroleptic malignant syndrome.

To decrease the physical risks of therapeutic seizures in ECT, general anesthesia is induced with an intravenous agent and muscle relaxant combined with bag-facemask ventilation for respiratory support during the resulting apneic period [2, 3]. Preoxygenation with 100% oxygen is also recommended by several guidelines to fill the oxygen reservoir because cerebral oxygen consumption increases by almost 200% during ECT-induced seizures [4].

Evidence suggests that greater ECT stimulus intensity with respect to the seizure threshold leads to more effective treatment and faster recovery [5], although high stimulus intensity is directly related to an increase in cognitive side effects. However, the seizure threshold tends to increase to a variable degree and at a variable rate over the course of ECT; thus, seizures may eventually be missed or inadequate. Therefore, several methods of seizure enhancement, inducing decreasing the anesthetic dosage if the agent has anticonvulsive properties, hyperventilation, and administration of adenosine receptor antagonists, caffein, or theophylline, are commonly employed [6]. Among these, hyperventilation easily decreases the patient’s carbon dioxide level, resulting in reduced seizure threshold and increased seizure duration. However, standardized ventilation maneuvers are not specified in these guidelines [7] when ECT is performed under anesthesia,

Some authors have suggested that a laryngeal mask could be a first-line option to manage airway and ventilation in ECT because of its minimal leaks, better airway control, and better ventilation features [8, 9]. In practice, facemask ventilation is frequently used due to convenience but is often criticized because it cannot effectively provide airway security. Previously, we used a one-handed technique for bag-facemask ventilation in ECT, which might occasionally be less effective, especially in patients who are obese or edentulous, which can be attributed to the failure to maintain a seal and patent upper airway. Therefore, we recently adopted a two-handed technique depending on the anesthesia machine to provide pressure-controlled ventilation to support successful ECT procedures.

Although the minimum duration of the seizure required for optimal therapeutic effects has never been established, typical recommended minimal seizure durations have been 20–25 seconds for motor response and 25 seconds for ictal electroencephalogram (EEG) response. Recently, other features of the seizure, besides duration, have emerged as promising measures of seizure quality. For example, a greater regularity predominantly shows high amplitude spikes during the slow-wave phase, greater postictal EEG suppression, and higher activation of the sympathetic nervous system [10]. Based on these assessments, this study aimed to compare seizure quality in ECT between one-handed and two-handed facemask techniques.

Although major cardiovascular complications are relatively rare with ECT, they may occur in older patients and in those with underlying cardiovascular diseases. Seizure activity is characteristically associated with prolonged sympathetic discharge following initial parasympathetic discharge, resulting in tachycardia and hypertension. Thus, we also compared the acute cardiovascular response to ECT between both facemask techniques.

## Methods

### Patients

The inclusion criteria of this the study were as follows; patients aged ≥18 years who were referred to receive index ECT by their treating psychiatrist, those receiving thiopental alone as an anesthetic (without adjacent anesthetic regimens, such as propofol, ketamine, remifentanil, or vasoactive drugs). The exclusion criteria were pregnancy, chronic obstructive pulmonary disease, bronchial asthma, abuse of alcohol or other toxic substances, inability to cooperate, and current major neurological illness or injury. The patients were 60 Japanese in-patients who underwent ECT at NHO Kure Medical Center, Japan. Patients were classified into two groups according to the facemask ventilation technique; the one-handed group (*n* = 30, between March 2020 and May 2020) and the two-handed group (n = 30, between March 2021 and June 2021). Written informed consent was obtained from all patients. Demographic, clinical, and infusion data were prospectively entered into the Department of Anesthesiology Information System and were retrospectively analyzed. The Institutional Review Board of the NHO Kure Medical Center approved the retrospective analysis of clinically acquired data and this study was approved by the Ethics Committees (2021–15, UMIN Clinical Trial Registry 000046544, date of registration 05/01/2022).

### Electroconvulsive therapy and medication

#### Preoxygenation, administration of anesthesia, and hyperventilation

The treatment sites were the ECT suite of the psychiatric inpatient ward for the one-handed group and the operating suite for the two-handed group. Concomitant psychotropic medications were not withdrawn during ECT. All patients were free of benzodiazepine use for at least 12 h prior to ECT treatment. Hyperoxia and hypocapnia during ECT help increase seizure quality in terms of intensity and duration. The patients were preoxygenated with 100% oxygen at 6 L/min via facemask during spontaneous ventilation before the induction of general anesthesia, with the goal of maintaining oxygen saturation at nearly 100%. The facemask was connected to an anesthesia machine (Excel 80™, Ohmeda, USA) in the one-handed group and to another anesthesia machine (Aespire™, GE Healthcare, USA) in the two-handed group, making the patients hyperventilate. Anesthesia was induced with intravenous thiopental (2–4 mg/kg). Before administrating the muscle relaxant, a tourniquet was placed on the ankle to allow visual and tactile observation of the motor component of seizure activity in that foot. Succinylcholine (1–2 mg/kg) was administered for muscle relaxation. Muscle relaxation was monitored using a peripheral nerve stimulator. In the one-handed group, the facemask was usually held with the left hand, and the right hand was freely used to provide patients with manual ventilation using a breathing bag. The number of insufflations and ventilation volumes were arbitrarily determined by each anesthesiologist. This technique was employed in the psychiatric ward.

In the two-handed group, pressure-controlled ventilation (positive inspiratory pressure, 20 cmH_2_O; ventilation rate, 25 breaths/min) was provided by an anesthesia machine in the operating suite. The left hand was positioned on one side of the facemask in the same way as in the one-handed technique, and the right hand was placed on the other side of the facemask in an identical conformation.

All procedures were conducted by the JSA Board Certified Anesthesiologists (JSA; Japanese Society of Anesthesiologists). Assisted ventilation was performed until the seizure ended and the patient recovered from anesthesia. Complete return of neuromuscular function allowing recovery from general anesthesia was ensured using quantitative monitoring of the degree of the residual blockade, with an adductor pollicis train-of-four ratio of at least 0.90.

Prior to discharge from the post-anesthetic recovery area, the patients were assessed using White’s fast-tracking score system. A score ≥ 12 (with no score < 1 in any individual category) and respiration rate ≥ 12 breaths/min were required before transfer to the inpatient floor.

#### Monitoring

During the ECT sessions, blood pressure, heart rate, oxygen saturation, electrocardiogram, and ventilation gases (oxygen and carbon dioxide) were recorded automatically before, during, and after the seizure. End-tidal carbon dioxide (EtCO_2_) monitoring was performed using a capnograph in the breathing circuit that was connected to the facemask. The tidal volume was obtained using a spirometer connected directly to the facemask via a heat and moisture exchanger.

#### ECT protocol

ECT was administered using a Thymatron^Ⓡ^ System IV (Somatics, LLC, USA) and brief pulse bi-frontal electrode placement in all patients. We used the stimulus program Low 0.5 of the device, which set pulse width at 0.5 ms and automatically adjusted the lowest frequency (max 70 Hz) for longest train duration (max 8.0 s) to achieve charge dose. The length and characteristics of the seizure were determined by two-channel EEG with electrodes at the Fp1 and Fp2 sites. Motor movement was monitored by an electromyogram using the isolated limb technique with a tourniquet.

#### Evaluation of seizure adequacy parameters

The typical recommended minimal durations were 20–25 seconds for the motor response and 25 seconds for the ictal EEG response. Ictal and peri-ictal EEG parameters, such as regularity and postictal suppression, were manually rated using standard methods from the literature [11–13]. Ictal regularity expressed global seizure strength on a seven-point scale ranging from 0 to 6 (regularity ≥5 defined as a regular slow wave). Higher numbers indicate an increasing regularity of ictal EEG recordings. Postictal suppression was defined as the degree of postictal bioelectric suppression on a four-point scale ranging from 0 to 3 (postictal suppression ≥2, designated as good seizure suppression).

The ECT stimulus and subsequent seizures both exert cardiovascular effects. First, the parasympathetic system is activated, leading to a decrease in blood pressure and heart rate at the end of the clonic phase. These events are followed by sympathetic hyperactivity, which in contrast, increases blood pressure and heart rate. The activation of the sympathetic nervous system was rated from 0 (poor) to 1 (good) (1 means increases in both blood pressure and heart rate which indicates adequate seizures).

#### Assessment of cardiovascular response

The cardiovascular response of patients was monitored before anesthesia, at the end of ECT treatment, and in the recovery settings, including blood pressure, heart rate, electrocardiogram, and peripheral oxygen saturation (SpO_2_).

### Statistical analysis

Data are presented as the median (IQR, 25–75% interquartile range) and mean ± standard deviation. Statistical analysis was performed using EZR (Saitama Medical Center, Jichi Medical University, Saitama, Japan), a graphical user interface for R (The R Foundation for Statistical Computing, Vienna, Austria). More precisely, it is a modified version of the R commander designed to add statistical functions frequently used in biostatistics [14].

The Mann–Whitney U test and Wilcoxon signed-rank test were used to compare variables, as appropriate. Categorical variables were compared using the Fisher’s exact test. Statistical significance was set at *p* <  0.05.

## Results

**Fig. 1:**
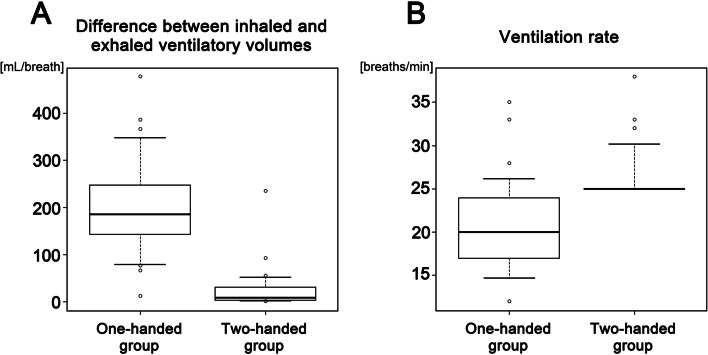
Ventilatory volumes and ventilation rate. **A** In the one-handed group, a greater difference between inhaled and exhaled ventilatory volumes suggests that a substantial volume leaked around the facemask during expiration. **B** Ventilation rate varies widely in the one-handed group

**Fig. 2:**
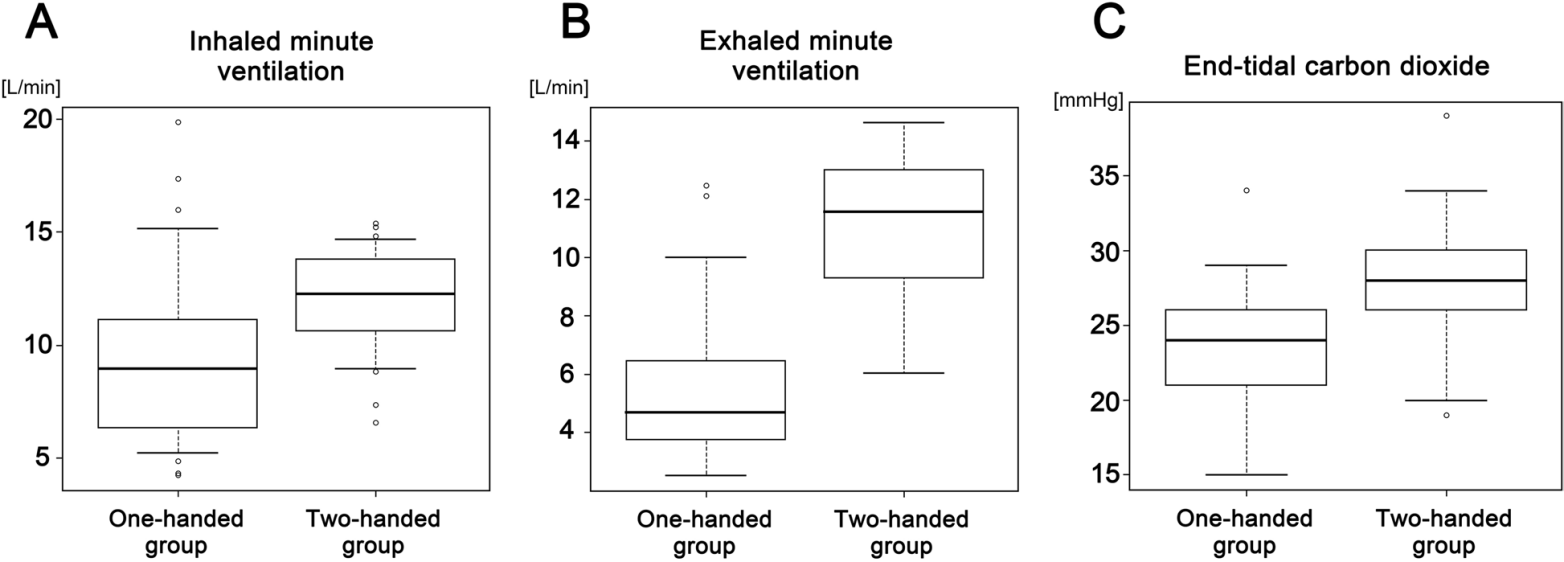
Minute ventilation and end-tidal carbon dioxide. **A** The two-handed group showing greater inhaled minute ventilation. **B** The two-handed group showing greater exhaled minute ventilation. **C** The lower end-tidal carbon dioxide (EtCO_2_) in the one-handed group might be uncertain due to greater leaks around the facemask during expiration. The EtCO_2_ value could be reliable in the two-handed group because of its minimal leaks

Patient demographics showed no significant difference in sex, age, height, body weight, or body mass index between the one-handed and two-handed groups (Table 1). There was no difference in the dose of thiopental administered during the induction of general anesthesia between the two groups (Table 1). Inhaled ventilatory volume did not differ between the one-handed and two-handed groups, whereas exhaled ventilatory volume was significantly lower in the one-handed group than in the two-handed group, resulting in a greater difference between inhaled and exhaled ventilatory volumes in the one-handed group (Fig. 1A). These data suggest that the one-handed technique produced a substantial volume of leaks around the facemask. The ventilation rate in the one-handed group was significantly lower than that in the two-handed group and varied widely among patients, whereas the ventilation rate in the two-handed group was set at 25 breaths/min with assisted pressure-controlled ventilation (Fig. 1B). Accordingly, the two-handed group showed greater inhaled and exhaled minute ventilation than the one-handed group (Fig. 2A, B). Nevertheless, EtCO_2_ was substantially lower in the one-handed group, which seemed to contradict its poor inhaled ventilation. This discrepancy may arise due to the intrusion of external air accompanying greater leaks around the facemask during expiration in the one-handed group; therefore, these EtCO_2_ data were not allowed to detect CO_2_ release at the end of the exhaled breath or did not properly represent hypocapnia arising from hyperventilation in the one-handed group. In the two-handed group, EtCO_2_ of approximately 30 mmHg was reliable because of the minimal leaks shown in this study (Fig. 2C).

**Table 1:** Clinical characteristics of patients and general anesthesia in the one-handed and two-handed groups

Variables	One-handed group	Two-handed group	***P*** value
Patients, n	30	30	
Male sex, n (%)	6 (20.0)	5 (16.7)	1.000
Age, years	64.1 ± 14.8	65.7 ± 12.9	0.836
Height, cm	154.9 ± 8.6	154.0 ± 7.2	0.807
Body weight, kg	54.4 ± 13.0	49.1 ± 10.0	0.129
Body mass index, kg/m^2^	22.5 ± 4.3	20.7 ± 3.9	0.093
**General anesthesia**			
Dose of thiopental, mg	133.3 ± 35.0	124.2 ± 38.5	0.262
Ventilatory volume during one breath, mL/breath			
Inhaled	460.7 ± 141.3	463.1 ± 102.2	0.416
Exhaled	259.0 ± 86.8	437.3 ± 100.9	< 0.001*
Difference between inhaled and exhaled ventilatory volumes, mL/breath	201.7 ± 98.6	25.8 ± 44.6	< 0.001*
Ventilation rate, breaths/min	20.6 ± 5.6	26.2 ± 3.0	< 0.001*
Minute ventilation, L/min			
Inhaled	9.52 ± 3.94	11.95 ± 2.29	< 0.005*
Exhaled	5.36 ± 2.46	11.29 ± 2.24	< 0.001*
Difference between inhaled and exhaled minute ventilations, L/min	4.16 ± 2.21	0.67 ± 1.14	< 0.001*
End-tidal carbon dioxide, mmHg	23.3 ± 4.4	28.2 ± 4.0	< 0.001*

Data are n (%) and mean ± standard deviation

*significant difference

In ECT, no significant difference was found in motor and seizure durations or stimulation doses between the two groups. Compared with the one-handed group, the two-handed group demonstrated better regularity in ictal EEG characteristics (Table 2).

**Table 2:** Ictal parameters and cardiovascular response in electroconvulsive therapy

Variables	One-handed group	Two-handed group	***P*** value
**Ictal parameters**			
Motor seizure duration, sec	35.1 ± 13.5	41.2 ± 14.7	0.233
EEG seizure duration, sec	67.5 ± 43.4	62.5 ± 30.7	0.865
Stimulation dose, %	60.8 ± 30.0	62.5 ± 27.1	0.952
Ictal EEG characteristics			
Regularity (0–6)	4 (3.75–5)	5 (5–6)	< 0.001*
Postictal suppression (0–3)	2 (2–2)	2 (2–2)	0.103
Activation of the sympathetic nervous system (0–1)	1 (1–1)	1 (1–1)	1.000
**Cardiovascular parameters**			
Baseline values before anesthetics			
SBP, mmHg	136.4 ± 19.9	142.3 ± 23.3	0.411
DBP, mmHg	74.5 ± 12.7	80.0 ± 11.1	0.057
MBP, mmHg	95.1 ± 13.6	100.8 ± 14.0	0.092
HR, bpm	98.0 ± 18.3	87.8 ± 22.3	0.065
SpO_2_, %	98.9 ± 1.7	97.7 ± 1.6	0.002*
ST, mV	0.045 ± 0.080	0.064 ± 0.062	0.493
After ECT seizure			
SBP, mmHg	194.1 ± 27.8	183.0 ± 27.1	0.262
DBP, mmHg	102.8 ± 23.2	103.9 ± 18.3	0.945
MBP, mmHg	133.2 ± 21.8	130.3 ± 19.7	0.622
HR, bpm	132.5 ± 17.0	121.2 ± 17.8	0.075
SpO_2_, %	95.2 ± 7.6	99.9 ± 0.3	0.024*
ST, mV	−0.033 ± 0.078	0.022 ± 0.063	0.030*
In the recovery area			
SBP, mmHg	149.1 ± 25.9	131.1 ± 16.5	0.027*
DBP, mmHg	75.4 ± 13.4	67.8 ± 7.9	0.080
MBP, mmHg	100.0 ± 16.5	88.9 ± 9.7	0.034*
HR, bpm	114.5 ± 16.6	91.6 ± 13.3	< 0.001*
SpO_2_, %	98.3 ± 1.8	99.5 ± 0.7	0.029*

Data are mean ± standard deviation and median (IQR; 25–75% interquartile range)

*significant difference, *ECT* Electroconvulsive therapy, *EEG* Electroencephalography, *SBP* Systolic blood pressure, *DBP* Diastolic blood pressure, *MBP* Mean blood pressure, *HR* Heart rate, *SpO*_*2*_ Peripheral oxygen saturation, *ST* ST-segment change on electrocardiogram in lead II

Before anesthesia, there was no significant difference in cardiovascular parameters between the two groups, except for SpO_2_, which decreased significantly in the two-handed group (Table 2). At the end of the ECT treatment, all parameters of blood pressure and heart rate increased significantly in both groups equally, with lower SpO_2_ and more ST-segment depression in the one-handed group. Comparing the baseline values before anesthesia, ECT treatment significantly depressed the ST-segment in both groups (one-handed group, − 0.078 ± 0.041 mV, *p* <  0.001; two-handed group, − 0.042 ± 0.020 mV, *p* <  0.001), while the degree of depression in the ST-segment increased significantly in the one-handed group (*p* <  0.001) compared to that in the two-handed group. In the recovery area, the one-handed group showed significantly higher blood pressure and heart rate, with lower SpO_2_ than the two-handed group. Following discharge from the recovery area, in the one-handed group two patients developed atrial fibrillation transiently and the other two patients had their SpO_2_ dropping to 89% on the inpatient floor.

## Discussion

Appropriate anesthesia is a critical part of successful ECT which is now a clinically established therapy for selection of psychiatric patients. This therapy is usually administered two or three times a week and can be extended up to 12 times as a course of one ECT treatment, depending on the individual’s continuing review. This ECT course is determined by psychiatrists in our institution. Since the seizure threshold gradually increases over the treatment course, a relatively high stimulation dose becomes necessary, which may result in a more rapid or effective treatment but can also possibly cause accentuation of cognitive disturbances. If the maximum stimulus intensity cannot create adequate seizures and they remain missed or inadequate, efforts should be directed at decreasing the seizure threshold, increasing seizure duration, or improving seizure quality [15]. First, hyperoxygenation decreases seizure threshold, increases seizure duration [16, 17] and amplitude, and causes postictal suppression [18]. Second, hypocapnia lowers the seizure threshold, prolongs seizure duration, and is correlated with some seizure quality indices [19]. Therefore, hypocapnia and hyperoxygenation elicit synergistically augmented effects [20].

Facemask ventilation is a straightforward, non-invasive airway management technique that is used as a primary mode of ventilation for short-duration anesthesia, such as ECT. Whether by one-handed or two-handed technique, 100% oxygen must be provided at an adequate flow rate to prevent rebreathing with no leaks around the facemask present to ensure adequate preoxygenation before induction of general anesthesia. In this study, we compared the effects of facemask ventilation between one-handed and two-handed techniques. In addition to fewer leaks around the facemask using the two-handed technique, a stable ventilation rate was previously determined and set with pressure-controlled ventilation, leading to greater inhaled minute ventilation in the two-handed group.

Because EtCO_2_ reflects the maximum CO_2_ concentration at the end of exhalation, which is close to the arterial CO_2_ value, EtCO_2_ monitoring is supposed to be a useful parameter of hyperventilation during ECT procedures [21]. Although there is still no consensus on the target EtCO_2_, excessive hypocapnia may increase the risk of cerebral and coronary vasoconstriction. By assessing ictal and postictal EEG parameters in the current study, both one-handed and two-handed techniques have a certain CO_2_ lowering effect because of an increase in seizure duration, a reduction in stimulus doses, and high numbers of ictal EEG characteristics. According to several studies, a target EtCO_2_ value of < 30 mmHg may not show any additional benefits of hyperventilation on seizure duration in ECT [9, 18, 21]. In particular, an EtCO_2_ of approximately 30 mmHg by the two-handed technique may be an ideal target value caused by hyperventilation because of the significantly higher regularity of ictal EEG characteristics. However, the use of a laryngeal mask may be a more effective approach because it demonstrated hyperventilation with higher ictal intensity or a higher postictal suppression index and amplitude in ECT [9]. However, routine use of this method for ECT is not currently recommended in clinical guidelines because of its invasive nature. In this study, one-handed ventilation induced lower EtCO_2_ levels compared with the two-handed technique, which can be explained by the intrusion of external air accompanying massive facemask leaks during expiration; thus, EtCO_2_ is ineffective in monitoring hypocapnia when using a one-handed technique.

We also explored the relationship between hyperventilation and hemodynamic parameters. ECT stimulus and the induced seizure both potentiate sympathetic discharge, which increases the heart rate and blood pressure, placing an increased demand on the cardiovascular system. Sudden onset of new changes in electrocardiograms after ECT might be reflective of myocardial ischemia due to myocardial oxygen consumption or non-uniform myocardial repolarization secondary to increased sympathetic activity due to massive release from the hypothalamus [22]. Coustet et al. demonstrated that acute hypoxia decreases the amplitude of the ST-segment in lead II [23]. The current study demonstrated that in both the one-handed and two-handed groups, ECT developed systemic hypertension and tachycardia to the same extent, suggesting the same level of postictal sympathetic agitation in these groups. Meanwhile, the one-handed group showed lower SpO_2_ and more ST-segment depression than the two-handed group, indicating that lower SpO_2_ induced by the one-handed technique subsequently elicited transient myocardial ischemia. In the recovery room, the one-handed group showed higher systolic pressure, tachycardia, and lower SpO_2_, which challenged the cardiovascular system, resulting in transient atrial fibrillation in two one-handed patients. The risks of cardiac arrhythmias, ischemia, and hypertension can be diminished by sufficient preoxygenation using the two-handed technique.

We demonstrated the efficacy of this two-handed technique during ECT. As a practical disadvantage, the two-handed technique depends on either an assistant or the use of pressure-controlled ventilation with an anesthesia machine to provide positive pressure ventilation. However, the use of pressure-controlled ventilation offers many advantages, including lower peak airway pressures and reduced inspiratory flow rates, which provide an additional safety measure against gastric insufflation compared with manual ventilation [24]. Since the 1960s, the complication rate of ECT has improved from 50% to almost anecdotal adverse events, similar to the morbidity and mortality rates observed in minor surgery. In the current study, the ECT procedures were performed in the psychiatric ward and the operating suite. The operating suite is superior to the psychiatric ward in terms of ensuring patient safety, such as patient monitoring and emergency response.

There are limitations of the trial that need to be addressed. First, the current study is the inability to draw clear conclusions regarding the effect of the two-hand technique on overall ictal parameters except for regularity. This study was a single-center investigation, and our dataset was a collection of response or observations from the entire population in both groups. Due to the small number of patients, the analysis might have been underpowered to detect differences in seizure quality and side effects. The main reason for the small number was to limit the patients to those receiving thiopental alone as an anesthetic. While we used thiopental as the primary anesthetic agent in ECT, other anesthetics were selected as alternative, including propofol, the combination of propofol and remifentanil, and ketamine, in patients with compromised pulmonary status or medical complications after earlier courses of anesthesia or ECT. Various anesthetics induce different advantages and disadvantages in ECT, which makes it difficult to compare the effects of one- and two-handed techniques among patients with other anesthetics. The protocols for other anesthetics may be assessed separately. Second, this study did not include data on psychiatric outcomes or cognitive side effects. As most clinical symptoms varied on a weekly basis, the effect of ECT was not always clarified the day after treatment. Thus, it is not clear whether the response to ECT by the two-handed technique is high enough to allow for fewer treatments. Further prospective research is needed to confirm the utility of the two-handed technique in ECT safety management. Third, we used pressure-controlled ventilation in the two-handed technique. Pressure control can change the tidal volumes depending on the patient’s lung compliance, resulting in variations in both ventilatory volume during one breath, and minute ventilation. As volume control attempts to maintain ventilation by increasing pressure, it can result in the movement of a large amount of air into the stomach if the airway is not opened properly, leading to vomiting and aspiration after ECT. In pressure-controlled ventilation, the setting of inspiratory pressure, and therefore, lower peak pressure, is prospective against barotrauma and gastric insufflation.

## Conclusions

In this study, we investigated methods to improve protocolized hyperventilation using a facemask in ECT. The two-handed technique with minimum facemask leaks is more effective at allowing hyperventilation than one-handed ventilation, leading to better EEG regularity. While, the two-handed group with sufficient preoxygenation did not cause more cardiovascular stress than the one-handed group. Furthermore, EtCO_2_ can effectively monitor hyperventilation in two-handed facemask ventilation systems.

## Data Availability

The original contributions presented in the study are included in the article, further inquiries can be directed to the corresponding author.

## Abbreviations

DBP: Diastolic blood pressure
ECT: Electroconvulsive therapy
EEG: Electroencephalography
EtCO_2_: End-tidal carbon dioxide
HR: Heart rate
MBP: Mean blood pressure
SBP: Systolic blood pressure
SpO_2_: Peripheral oxygen saturation
